# Comparative In Vitro Immune Stimulation Analysis of Primary Human B Cells and B Cell Lines

**DOI:** 10.1155/2016/5281823

**Published:** 2016-12-26

**Authors:** Kristien Van Belle, Jean Herman, Louis Boon, Mark Waer, Ben Sprangers, Thierry Louat

**Affiliations:** ^1^KU Leuven, Interface Valorisation Platform (IVAP), 3000 Leuven, Belgium; ^2^Department of Microbiology & Immunology, Laboratory of Experimental Transplantation, KU Leuven, 3000 Leuven, Belgium; ^3^Department of Pediatric Nephrology and Solid Organ Transplantation, University Hospitals Leuven, 3000 Leuven, Belgium; ^4^Bioceros, 3584 CM Utrecht, Netherlands; ^5^Department of Nephrology, University Hospitals Leuven, 3000 Leuven, Belgium

## Abstract

B cell specific immunomodulatory drugs still remain an unmet medical need. Utilisation of validated simplified in vitro models would allow readily obtaining new insights in the complexity of B cell regulation. For this purpose we investigated which human B lymphocyte stimulation assays may be ideally suited to investigate new B lymphocyte immunosuppressants. Primary polyclonal human B cells underwent in vitro stimulation and their proliferation, production of immunoglobulins (Igs) and of cytokines, and expression of cell surface molecules were analysed using various stimuli. ODN2006, a toll-like receptor 9 (TLR9) agonist, was the most potent general B cell stimulus. Subsequently, we investigated on which human B cell lines ODN2006 evoked the broadest immunostimulatory effects. The Namalwa cell line proved to be the most responsive upon TLR9 stimulation and hence may serve as a relevant, homogeneous, and stable B cell model in an in vitro phenotypic assay for the discovery of new targets and inhibitors of the B cell activation processes. As for the read-out for such screening assay, it is proposed that the expression of activation and costimulatory surface markers reliably reflects B lymphocyte activation.

## 1. Introduction

Current immunotherapeutic drugs have improved the life expectancy of patients, but they still exhibit important side effects. Furthermore, the number of new immunotherapeutic small molecule medicines and biologicals entering clinical development is in decline despite increasing levels of investments in the drug industry [1–3]. Moreover, the majority of the marketed immunotherapeutic drugs are focused on controlling the activity of T cells (e.g., calcineurin inhibitors [cyclosporine A, tacrolimus]; mTOR inhibitors [sirolimus, everolimus]; costimulation blocking antibodies [belatacept, abatacept]; CD3 antagonistic antibody [muromonab]; or CD25/IL2-R antagonistic antibodies [basiliximab, daclizumab]). Nevertheless, B cells are equally important players in the immune response, but presently there are only very few drugs available to target them. The effector functions of B cells are diverse. Production of Igs assures the clearance of invading pathogens and dying cells [4, 5]. B cells are efficient antigen-presenting cells capturing antigen with their antigen-specific B cell receptor (BCR) and presenting the epitopes, bound to major histocompatibility complex (MHC) molecules, to the appropriate T cells. Through the secretion of cytokines [6, 7] and the expression level of various cell surface markers, activated B cells can establish an effective intercellular communication with other effector cells to obtain a more directed and controlled immune response. The strength of the B cell lies not only in its versatility of actions, but also in its ability to adapt its phenotype in response to (micro)environmental variables. B cells play a considerable, but not yet fully understood, role as a pathogenic factor in different clinical situations such as cancer [8], autoimmune disorders [9–11], transplant rejection [12–16], and graft-versus-host diseases [17–19].

At the present time, there are only very few B cell specific immunomodulatory agents (e.g., bortezomib, rituximab, and belimumab) available in the clinic and they are mainly depleting agents. Hence, there is an unmet need for new drugs in this field.

Exploration of B cell regulation models could lead to the identification of relevant new targets or molecular agents with potential as B cell drugs. The goal of the present study was to investigate a series of B cell stimuli and human B cell lines to identify an in vitro model which is suitable to explore B cell immune activation and readily applicable for screening and drug development.

## 2. Materials and Methods

### 2.1. Cell Culture Media

Complete RPMI 1640 culture medium consisted of RPMI 1640 with 10% foetal calf serum (FCS, HyClone® Thermo Scientific, United Kingdom) and 5 *μ*g/mL gentamicin sulphate antibiotics. Complete DMEM culture medium consisted of DMEM (Dulbecco's Modified Eagle's Medium) with 10% heat-inactivated FCS and 5 *μ*g/mL gentamicin sulphate antibiotics. Cell culture media and gentamicin sulphate antibiotics were purchased from BioWhittaker® Lonza (Verviers, Belgium).

### 2.2. Cells and Cell Lines

Blood samples of healthy volunteers were collected at the Red Cross of Mechelen, Belgium. Each donor consents to the use of his blood for research purposes. Human peripheral blood mononuclear cells (PBMCs) were obtained by density gradient centrifugation of the heparinized venous blood over Lymphoprep™ (Axis Shield PoC AS; density 1,077 ± 0.001 g/mL). Highly purified naive peripheral human B cells were separated from fresh human PBMCs on magnetic columns by positive selection using CD19 magnetic beads according to the manufacturer's instructions (MACS Miltenyi Biotech, Leiden, Netherlands). The purity of the isolated naive B cells was ≥95% as analysed by flow cytometry. Cells were suspended at the desired concentration in complete culture medium. Human B cell lines Daudi, Raji, Ramos (Deutsche Sammlung von Mikroorganismen und Zellkulturen, DSMZ, Germany), Namalwa, RPMI 8866 (European Collection of Cell Cultures, ECACC, England), and RPMI 1788 (Global Bioresource Center ATCC, USA) were used. The cell lines were maintained in culture flasks (TPP, Switzerland) as suspension cultures in complete RPMI 1640 culture medium at 37°C and 5% CO_2_.

### 2.3. Pharmacological Agents

Pharmacological inhibitors AZD-5363, ibrutinib, and MK-2206.2HCl were purchased from SelleckChem (Munich, Germany); chloroquine, LY-294,002, Mirin, SAHA, and TPCA-1 from Sigma-Aldrich (Diegem, Belgium); STAT3 inhibitor VII from Calbiochem, Merck Millipore (Overijse, Belgium); bortezomib and dasatinib from LC Laboratories (Woburn, MA, USA).

### 2.4. In Vitro Stimulatory Conditions

Human primary B cells were stimulated with a variety of conventional in vitro stimulatory conditions. The following stimulatory reagents were used: oligodeoxynucleotide 2006 (ODN2006, InvivoGen, Toulouse, France); resiquimod, lipopolysaccharide (LPS), and pokeweed (Sigma-Aldrich, Diegem, Belgium); anti-CD40 (Bioceros BV, Utrecht, Netherlands); anti-IgM (Jackson ImmunoResearch, Suffolk, United Kingdom); pansorbin (Calbiochem, Merck Millipore, Overijse, Belgium); 2,4,6-trinitrophenyl hapten conjugated to bovine serum albumin (TNP-BSA) and 2,4,6-trinitrophenyl hapten conjugated to Ficoll (TNP-Ficoll) (Biosearch technologies, Novato, California, USA); recombinant human IL4 and IL21 (MACS Miltenyi Biotec, Leiden, Netherlands); recombinant human IL2 and IL10 (BioLegend, ImTec Diagnostics NV Antwerp, Belgium). Primary B cells were stimulated with the reagents at different concentrations and the most optimal reagent concentrations were then used for further experiments. Following in vitro stimulatory conditions were applied in duplicate: 0,1 *μ*M ODN2006; 1 *μ*M resiquimod; 1 *μ*g/mL LPS; 5 *μ*g/mL anti-CD40 alone or in combination with 20 ng/mL IL4 or with 12.5 ng/mL IL21; 5 *μ*g/mL anti-IgM with or without 20 ng/mL IL4; 1/10 000 pansorbin with 100 units/mL IL2 and 50 ng/mL IL10; 1 *μ*g/mL pokeweed; 91 *μ*g/mL TNP-BSA; or 91 *μ*g/mL TNP-Ficoll. Human cell lines were stimulated for 24 hours in presence of 0.1 *μ*M ODN2006.

### 2.5. Ig Production

For assessment of Ig production, freshly isolated human CD19^+^ B cells were plated at 25 000 cells per well in a 384-well plate (Perkin Elmer, Zaventem, Belgium) in 55 *μ*L complete DMEM medium. After 7 days of stimulation, supernatant was taken for analysis of IgG and IgM with the AlphaLISA human IgG and IgM kits according to the manufacturer's instructions (Perkin Elmer, Zaventem, Belgium). Analysis was performed with the EnVision™ 2103 Multilabel Reader (Perkin Elmer, Zaventem, Belgium). For IgG production, less than 5-fold increase was considered as a weak effect of the stimulus, 5- to 20-fold increase as a moderate effect, and more than 20-fold increase as a strong effect. For IgM production, less than 5-fold increase was considered as a weak effect of the stimulus, 5- to 10-fold increase as a moderate effect, and more than 10-fold increase as a strong effect.

### 2.6. Cytokine Production

Freshly isolated human CD19^+^ B cells were plated at 50 000 cells per well in a 96-well plate in 220 *μ*L complete DMEM medium and activated by different stimulatory conditions. Analysis of IL6 and IL8 was performed after 2 days with the AlphaLISA human IL6 and IL8 kits according to the manufacturer's instructions (Perkin Elmer, Zaventem, Belgium). Analysis was performed with the EnVision 2103 Multilabel Reader (Perkin Elmer, Zaventem, Belgium). For IL6 production, less than 5-fold increase was considered as a weak effect of the stimulus, 5- to 20-fold increase as a moderate effect, and more than 20-fold increase as a strong effect. For IL8 production, less than 5-fold increase was considered as a weak effect of the stimulus, 5 to 10-fold increase as a moderate effect, and more than 10-fold increase as a strong effect.

### 2.7. B Cell Proliferation

Freshly isolated human CD19^+^ B cells were plated at 50 000 cells per well in a 96-well plate in 220 *μ*L complete DMEM medium and activated by different stimulatory conditions. Ten *μ*Ci ^3^H thymidine (Perkin Elmer, Zaventem, Belgium) was added in the wells for the last 18 hours of 3 days of incubation. The cells were harvested on glass filter paper in 96-well format (Perkin Elmer, Zaventem, Belgium). After drying, radioactivity was counted in a scintillation counter (TopCount, Perkin Elmer, Zaventem, Belgium). Less than 5-fold increase in proliferation was considered as a weak effect of the stimulus, 5 to 20-fold increase as a moderate effect, and more than 20-fold increase as a strong effect.

### 2.8. Flow Cytometry

Freshly isolated human CD19^+^ B cells and cells of human B cell lines were plated at 50 000 cells per well in a 96-well plate in 220 *μ*L complete DMEM medium and analysed for their cell surface markers by a 3-color Becton Dickinson FACSCalibur apparatus after 24 hours of stimulation as described above. Fluorescein isothiocyanate (FITC), phycoerythrin (PE), or phycoerythrin-cyanine 5- (Pe-Cy5-) labelled antibodies to CD40, CD69, CD70, CD80, CD83, CD86, MHC class I, and MHC class II were purchased from BioLegend (ImTec Diagnostics NV, Antwerp, Belgium). Cells were washed twice with cold phosphate buffered saline (PBS) and then incubated 30 minutes at 4°C with fluorochrome-conjugated antibodies diluted in cold PBS. After incubation and two washing steps with cold PBS, cells were suspended in PBS with 2% paraformaldehyde and analysed. Less than 1.5-fold increase in mean fluorescence intensity (MFI) was considered as a weak effect of the stimulus, 1.5- to 2-fold increase was considered as a moderate effect and more than 2-fold increase as a strong effect.

### 2.9. Human Mixed Lymphocyte Reaction

Freshly isolated human PBMCs were the responder cells and growth-inhibited RPMI 1788 cells served as stimulator cells in the human mixed lymphocyte reaction (MLR) assay. To block their proliferation RPMI 1788 cells were treated with 96 nM mitomycin C (mitomycin C Kyowa®, Takeda Belgium, Brussels, Belgium) for 20 minutes at 37°C. After three washes with RPMI 1640 containing antibiotics, the stimulator cells were then diluted at the desired cell concentration in complete RPMI 1640 medium. The responder human PBMCs were cocultivated with the stimulator cells, ratio responder/stimulator of 8/3, in complete RPMI 1640 culture medium for 6 days at 37°C and 5% CO_2_. DNA synthesis of the responder cells was assayed by the addition of 10 *μ*Ci ^3^H thymidine (Perkin Elmer, Zaventem, Belgium) during the last 18 hours of culture. The cells were harvested on glass filter paper in 96-well format (Perkin Elmer, Zaventem, Belgium). After drying, radioactivity was counted in a scintillation counter (TopCount, Perkin Elmer, Zaventem, Belgium).

### 2.10. WST-1 Viability Assay

Analysis of cytotoxic and cytostatic compounds on Namalwa cell line was done with the WST-1 viability assay. The cell proliferation reagent WST-1 (Roche Diagnostics, Mannheim, Germany), a soluble tetrazolium salt, was used for the spectrophotometric quantification of cell proliferation and viability in cell populations using the 96-well plate format. Quantification of the formed formazan was done by a scanning multiwall spectrophotometer. Compounds were added at different doses to the cell lines. After 48 hours of incubation at 37°C and 5% CO_2_, Triton® X-100 (0.5% final; Fluka Biochemika) was added in the control wells and 10 *μ*L of WST-1 reagent was added in each well. Cells were incubated for 2 to 4 hours at 37°C and 5% CO_2_. The absorbance of the formazan dye was measured by the EnVision 2103 Multilabel Reader (Perkin Elmer, Zaventem, Belgium) at 540 nM.

### 2.11. Statistical Analysis

Comparison of activation marker upregulation on B cells versus Namalwa was performed by* t*-test analysis of induction ratios. *p* values less than 0.05 are considered as significant.

## 3. Results

### 3.1. In Vitro Immune Stimulation

B cells act in a specific manner according to the nature and the strength of the stimulatory signal they receive. Natural stimulatory conditions in vivo can be simulated in vitro. Several in vitro stimulatory conditions were tested on purified human primary B cells in order to find the stimulus that induces the clearest and broadest immunostimulatory effects.

#### 3.1.1. Phenotypic Outcome of Various In Vitro Stimulatory Conditions on Primary Human B Cells

Table 1 gives an overview of different stimuli tested on primary human B cells and their effect on various phenotypic responses at different time points after initiation of the stimulation. Stimulation of B cells with the hapten-modified T-independent antigen TNP-Ficoll had neither effect on proliferation and production of Igs or cytokines nor on the expression of cell surface markers.

TNP-BSA, considered as a T cell/CD40L dependent stimulus, resulted in moderate induction of B cell proliferation and of Ig and IL6 production. The production of IL8 was more strongly induced. Both CD69 and MHC class I expression showed a moderate augmentation following TNP-BSA stimulation.

Anti-IgM antibodies elicit aggregation of the BCR as it happens in vivo after antigen ligation. On their own, anti-IgM antibodies proved to be weak inducers of in vitro B cell activation. However, in combination with IL4 there was a high proliferation rate and IL6 production; production of IgG augmented and activation markers CD69 and CD83 were strongly upregulated whereas the expression of CD40 was only moderately increased. Besides this, there was no or only a limited effect of anti-IgM with IL4 on the other cell surface markers. The production of IgM could not be measured when anti-IgM antibodies were used for the stimulation of human B cells.

Agonistic anti-CD40 antibodies mimic the ligation CD40-CD40L which stimulates the clonal expansion and differentiation of B cells. The agonistic anti-CD40 antibodies we used in the assays were by themselves only a weak stimulus for primary B cells with poor effects on proliferation, Ig, and cytokine production. However, when combined with IL4 or IL21, moderate to strong effects on proliferation, Ig production, and expression of several cell surface markers were obtained. The expression of CD40 could not be analysed due to the agonistic anti-CD40 antibodies used for the stimulation. The secretion of IL6, but not of IL8, was strongly increased with the combination of agonistic anti-CD40 antibodies with IL4. IL21, which is a very potent inducer of terminal B cell differentiation in humans [20], increased by itself strongly the expression of CD69, and was a moderate inducer of IgG and IgM production. There was no effect recorded on cytokine production nor on the expression of the other cell surface markers.

Next, three TLR agonistic ligands were tested for their potential as B cell stimulator. LPS is the major component of the outer membrane of Gram-negative bacteria and is a ligand of TLR4. LPS did not show any effect, besides a small increase of IL6 and IL8. This unresponsiveness was not unexpected, because human B cells, unlike murine B cells, lack significant TLR4 expression [21]. The small effect on IL6 and IL8 is probably due to an effect on the very small percentage of contaminating non-B cells.

Resiquimod belongs to the class of imidazoquinolinamines and is a synthetic agonist of TLR7/TLR8. It turned out to be a potent B cell activator with strong effect on the expression of various activation and costimulatory cell surface markers and on the production of IgM, IL6, and IL8. Only a moderate effect was observed on cellular proliferation and on IgG production.

ODN2006 belongs to the class B CpG ODNs which are synthetic oligonucleotides which contain unmethylated CpG dinucleotides in particular sequence contexts (CpG motifs) and are recognized by human TLR9. ODN2006 showed more potency than resiquimod in the cellular proliferation and in the IgG production. However, the production of IL8 was less apparent than with resiquimod. Most cell surface markers were strongly upregulated upon stimulation with ODN2006.

Pansorbin cells are heat-killed, formalin-fixed* Staphylococcus aureus* cells which have a coat of protein A and can activate B cells through cross-linking of surface Igs [22]. In combination with IL2 and IL10, pansorbin provoked a strong boost in B cell proliferation and in Ig and cytokine production. There were no effect on the markers CD40 and MHC class I and class II and a moderate effect on CD80, but expression of CD69, CD70, CD83, and CD86 was strongly elevated.

Pokeweed mitogen is a carbohydrate-binding lectin, isolated from the pokeweed plant* Phytolacca americana*, with stimulatory effects on B cells presumably attributed to the cross-linking of glycoproteins on the B cell surface [23]. Pokeweed showed stimulatory effect on B cell proliferation and on the production of IgM, IL6, and IL8. But IgG production was not induced and expression of the cell surface markers hardly changed.

From all the stimulation conditions tested, ODN2006 was selected as the stimulus of choice, because it is able to induce B cell proliferation, IgM and IgG secretion, cytokine release, and activation markers upregulation.

#### 3.1.2. Comparative Analysis of Various Human B Cell Lines in Their Cell Surface Marker Expression after ODN2006 Stimulation

Primary B cells are not convenient for repetitive assays because of the disparity between the different human blood donors and because of the heterogeneity of B cell subpopulations after isolation and purification of the B cells from peripheral blood. Using a homogeneous, immortalized B cell line would circumvent many of these drawbacks. As ODN2006 appeared to have the most broad stimulatory effect on polyclonal B cells, six different human B cell lines, Daudi, Namalwa, Raji, Ramos, RPMI 1788, and RPMI 8866, were investigated for their reactivity upon in vitro ODN2006 stimulation. Since, depending on their development stage, cancerous B cell lines frequently display aberrancies in Ig and cytokine production, the focus was placed on the expression of the cell surface markers.

**Figure 1:**
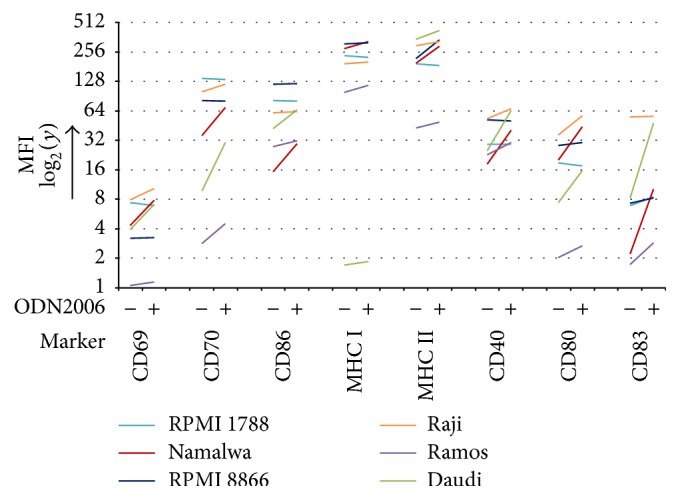
Flow cytometry analysis of cell surface markers on human B cell lines after stimulation with 0.1 *μ*M ODN2006 for 24 hours. This graph is a representative of three independent experiments. Each colored line in the graph represents a human B cell line and displays the change in expression (MFI, *y*-axis) of the different cell surface markers (marker, *x*-axis) between naive (“−,” *x*-axis) and ODN2006-stimulated (“+,” *x*-axis) cells.

The six human B cell lines were evaluated by flow cytometry for their expression of CD40, CD69, CD70, CD80, CD83, CD86, and MHC class I and class II before and after 24 hours of stimulation with the TLR9 agonist ODN2006. Results are presented in Figure 1. Stimulation of both RPMI 1788 and RPMI 8866 with ODN2006 had hardly any effect on all investigated markers. Between the four Burkitt lymphoma cell lines Daudi, Namalwa, Raji, and Ramos, Daudi and Namalwa showed a very dynamic response for most of the markers after ODN2006 stimulation. Daudi, however, did not express the marker MHC class I; therefore Namalwa was chosen as our cell line model of B cells.

**Figure 2:**
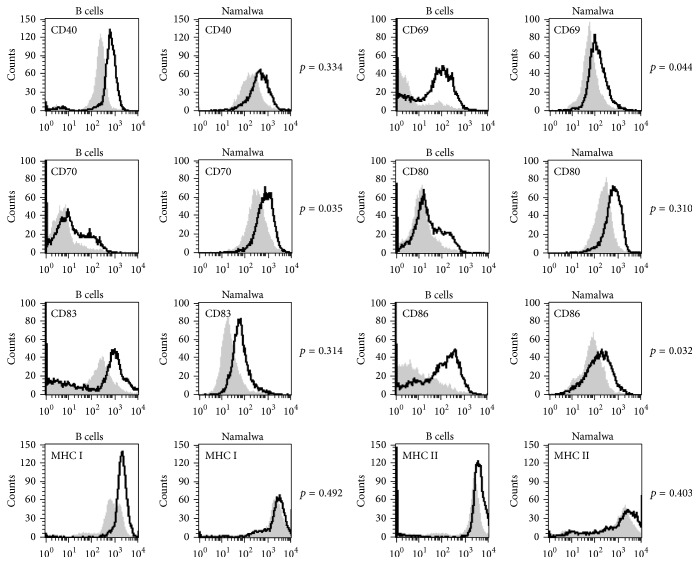
Comparative flow cytometric analysis of cell surface markers shows a close resemblance in expression profile between human peripheral B cells and Namalwa. The histogram plots from one out of four independent experiments show the expression of the cell surface markers before (grey region) and after 24 hours of stimulation (black line) with 0.1 *μ*M ODN2006. Comparison of activation marker upregulation on B cells versus Namalwa was performed by* t*-test analysis of induction ratios for each marker from four independent experiments.

Next, it was investigated if, for the same ODN2006 stimulation, Namalwa behaved as polyclonal human B cells (Figure 2) and could be used as a reliable model. For some of the expression markers (CD40, CD83) expression was homogeneous in both polyclonal and Namalwa B cells and clearly further increased after ODN2006 stimulation. Other markers (CD69, CD70, CD80, and CD86) were much less homogeneous at baseline in polyclonal than in Namalwa B cells but did in both cases clearly increase after ODN2006 stimulation. For CD69 and CD86, the induction was more pronounced in B cells than in Namalwa cells (CD69: 6.4-fold for B cell versus 2.0 for Namalwa, *p* value 0.044, CD86: 5.2-fold versus 2.0, resp., *p* value 0.032). On the opposite, CD70 was more induced on Namalwa cells than on B cells (2.1-fold versus 1.8, *p* value 0.035). As for MHC antigen expression, this was generally very high in Namalwa and B cells at basal level precluding further strong increase after ODN2006 stimulation. All together Namalwa and polyclonal B cells show a similar pattern in the induction of activation markers after TLR9 stimulation.

### 3.2. Characterization of Immunosuppressive Drugs or New Inhibitors Using ODN2006-Stimulated Namalwa Cells or Primary B Cells

To verify if we could detect B cell immunosuppressants through the model of ODN2006-stimulated Namalwa and to determine if there is a consensus between the polyclonal primary B cell Ig production and the expression of activation and costimulatory markers on Namalwa, some established pharmacological agents were investigated simultaneously in functional assays (MLR, B cell IgG secretion assay) and in flow cytometric assay of markers on ODN2006-stimulated Namalwa, a cytotoxicity counterscreen being performed on Namalwa (Table 2).

Both bortezomib, a selective small molecule inhibitor of the mammalian 26S proteasome, and dasatinib, an inhibitor of multiple tyrosine kinases, were cytotoxic on Namalwa cells as shown by the very low IC_50_-values in the WST-1 viability assay. This precluded further examination in functional B cell assays.

Ibrutinib, a selective Bruton's-tyrosine kinase (BTK) inhibitor, used in clinic as an anticancer drug targeting B cell malignancies, impeded very potently IgG production by ODN2006-stimulated B cells but showed much less effect in MLR. All cell surface markers, except for CD69 and CD80, were significantly downregulated by ibrutinib at nontoxic concentration.

Chloroquine is a weak base which accumulates in acidic compartments, like the endosomes, and inhibits TLR9 signalling by preventing its cleavage and activation. It reduced strongly the production of IgG by peripheral B lymphocytes and decreased to a similar extent the expression of activation and costimulatory markers on Namalwa cells.

LY-294,002, a phosphatidylinositol-4,5-bisphosphate 3-kinase (PI3K) inhibitor, inhibited the production of IgG of polyclonal B cells and the expression of CD86 on Namalwa confirming the involvement of PI3K in B cell activation processes.

MK-2206.2HCl and AZD-5363, both AKT1/2/3 inhibitors, inhibited IgG production by peripheral B cells and suppressed overexpression of CD40, CD70, CD80, CD83, and CD86 upon ODN2006 activation on Namalwa cells. In contrast, CD69 experienced a boost in expression induced by both agents. MK-2206 was more potent on IgG, CD80, and CD83 than AZD-5363 suggesting a difference in pharmacodynamics or in specificity-properties of the compound.

TPCA-1 is a human I*κ*B kinases (IKK) antagonist that impedes the nuclear localization of nuclear factor kappa-light-chain-enhancer of activated B cells (NF-*κ*B). It significantly decreased the expression of the activation and costimulatory markers on Namalwa cells and the IgG production by peripheral B cells. Nonetheless, TPCA-1 is not a B cell-selective modulator as it appeared to be a potent compound in MLR, conforming to NF-*κ*B also being described in T cell activation pathways.

STAT3 inhibitor VII, antagonist of transcription factor signal transducer and activator of transcription 3 (STAT3), demonstrated hardly any effect in the performed assays, except that it moderately impeded the upregulation of costimulatory marker CD86.

Suberoylanilide hydroxamic acid (SAHA) is a potent, reversible pan-histone deacetylase inhibitor. It inhibits both class I and class II HDACs, altering gene transcription and inducing cell cycle arrest and/or apoptosis in a wide variety of transformed cells. Although SAHA proved to be slightly cytotoxic for Namalwa and impeded IgG production, it did not show potency on the expression of cell surface markers. Similarly, Mirin, a DNA repair targeting agent showed an effect on IgG secretion by ODN2006 stimulated B cells but had no effect on activation markers on Namalwa cells after TLR9 stimulation.

Table 2 shows that IgG secretion in ODN2006 activated B cells and activation markers upregulation on ODN2006 stimulated Namalwa share several signalling pathways (NF-kB, tyrosine kinases, and serine/threonine kinases) while others are not shared (HDAC, DNA repair).

## 4. Discussion

As there is a strong unmet need to target B lymphocytes by specific immunosuppressive drugs, there is a similar need for a broad in vitro immunoassay that could simplify the screening of such potential agents. Although they have limitations, in vitro assays form an abstract approximation of the actual in vivo conditions and are essential to obtain insight into complex biological phenomena leading to new discoveries and predictions. That assay has to involve a stimulus which leads to broad phenotypic changes in a stable B cell model. For this purpose, we first compared 13 different stimuli for their capacity to activate human primary B cells by looking at several activation outcomes: cellular proliferation, IgM and IgG secretion, cytokine release, and upregulation of different activation markers. To the best of our knowledge this is the first report comparing these stimuli on several activation outcomes. Amongst the various stimuli investigated, in vitro stimulation with TLR9 agonist ODN2006 resulted in the “broadest” phenotypic change profile in human polyclonal B cells. The pattern recognition receptor TLR9 recognizes the CpG motifs in oligodeoxynucleotides as pathogen-associated molecular patterns due to their abundance in microbial genomes and their rarity in vertebrate genomes, except in the mitochondrial DNA [24]. TLR9 signalling mediates the activation of both innate and adaptive humoral and cellular immunity against viral and bacterial infections by promoting cellular proliferation and differentiation into antibody-secreting cells, upregulating molecules involved in immune cellular interactions, and increasing secretion of proinflammatory (IL6, TNF*α*, and type I interferons) and immune regulatory (IL10) cytokines [25, 26]. The molecular pathways triggered by TLR9 activation involved, as confirmed with the pharmacological agents we assessed, NF-*κ*B, PI3K, tyrosine, and serine/threonine kinases [27].

The heterogeneity between the different human blood donors, the heterogeneity after isolation and purification from peripheral blood, the limited yield of the purification, and the short longevity make primary B cells less optimal for repeatable assays. So a monoclonal population of dividing cells that could substitute the primary B cell would stabilise and facilitate the assay by overcoming the aforementioned limitations. The Namalwa cell line appeared as the best model to mimic the primary B cells when the expression of cell surface markers is used as read-out.

In vitro and in vivo studies have indicated that cell surface markers CD40, CD70, CD80, and CD86 are more than just costimulatory molecules for activation of CD4^+^ T cells. Indeed, they are also key molecules in the signalling for the regulation of Ig production, particularly of IgG. CD80/CD86 activation plays a key role in regulating the IgG1-production by previously activated B cells [28, 29]. Naive B cells from patients with common variable immunodeficiency are markedly impaired in upregulating the costimulatory molecules CD86 and CD70 upon BCR cross-linking and the expression remained reduced even in the presence of autologous helper CD4^+^ T cells. The insufficient upregulation of these two crucial costimulatory molecules could explain the poor class switching and, hence, reduced Ig serum levels, except for IgM [30, 31]. Similarly, inadequate CD40-CD40L interactions, as depicted in the X-linked immunodeficiency with hyper-IgM syndrome, cause defects in Ig class switching, a central process to antigen-dependent B cell maturation and to the generation of memory B cells and plasma cells [32].

Several pharmacological agents were assessed to challenge our assay in the prediction of B cell targeting agents. Cytotoxic compounds were identified by the Namalwa WST-1 viability assay (bortezomib and dasatinib) and excluded from further investigation. Compounds able to suppress overexpression of cell surface markers on Namalwa cells after the ODN2006 challenge in a range of concentration lower than the IC_50_ detected in the viability assay can be considered as hits. The costimulatory marker CD86 appears to be the most sensitive marker.

We confirmed the involvement of BTK, PI3K/AKT, and NF-*κ*B pathways in our assay. Inhibitors of these pathways were also able to block the production of IgG by human primary B cells after ODN2006 stimulation, confirming the suitability of the assay to detect inhibitors of IgG production. Chloroquine was also found to be a potent inhibitor both in the Namalwa activation markers assay and in the B cell IgG assay. This is not surprising as chloroquine is a very proximal inhibitor interfering with the first step of the TLR9 activation [33].

Some compounds active in the IgG production assay can be missed in our screening (false negatives), as illustrated by Mirin or SAHA. This can be explained by the fact that both compounds target the DNA recombination step during the Ig class switch, a process that does not occur in the Namalwa cell line that secretes constitutively IgM [34]. It must be realized that, as for many screening assays, results can be “falsely positive” and, hence, that a new compound may not work in primary human B cells, because the pathway it blocks in Namalwa may be not pertinent or may be bypassed in primary human B cells. To exclude “false positivity,” hits selected after the screening must be confirmed in other assays like the B cell IgG assay potentially with different stimuli to validate them as broad B cell inhibitors.

In conclusion, the limitations inherent to the use of human primary B cells in repetitive and large-scale experiments have been circumvented through the Namalwa B cell line. The described in vitro immunoassay with ODN2006-stimulated Namalwa cells and with flow cytometric read-out of the activation and costimulatory cell surface markers can serve as a potent and robust first-line screening to identify potential new B cell active compounds or to refine mechanisms of action of known immunomodulators.

## Figures and Tables

**Table 1:** Immune effects at various time points after initiation of stimulation.

Stimulus	Read-out												
	CD40	CD69	CD70	CD80	CD83	CD86	MHC I	MHC II	IL6	IL8	Proliferation	IgG	IgM
	24 hours								2 days		3 days	7 days	
TNP-Ficoll	+	+	+	+	+	+	+	+	+	+	+	+	+
TNP-BSA	+	++	+	+	+	+	++	+	++	+++	++	++	++
Anti-IgM	+	++	+	+	++	+	+	+	+	+	+	+	NA
Anti-IgM + IL4	++	+++	+	+	+++	+	+	+	+++	+	+++	++	NA
Anti-CD40	NA	++	+	+	++	++	+	+	+	+	+	++	+
Anti-CD40 + IL4	NA	+++	+	++	+++	+++	+	+	+++	+	+++	++	+++
Anti-CD40 + IL21	NA	+++	+	++	+	+++	++	+	+	+	+++	+++	+++
IL21	+	+++	+	+	+	+	+	+	+	+	+	++	++
LPS	+	+	+	+	++	+	+	+	++	++	+	+	+
Resiquimod	+++	+++	++	++	++	+++	+	+	+++	+++	++	++	+++
ODN2006	+++	+++	++	+++	+++	+++	++	+	+++	++	+++	+++	+++
Pansorbin + IL2 + IL10	+	+++	+++	++	+++	+++	+	+	+++	+++	+++	+++	+++
Pokeweed	+	+	+	+	+	+	+	+	++	+++	++	+	+++

In four independent experiments, different in vitro stimulatory conditions were tested on freshly isolated human B cells; the strength of their effect on proliferation, Ig and cytokine production, and expression of cell surface markers is represented in the table (+++ = strong effect, ++ = moderate effect, + = weak effect, and NA = not analysed). For the definition of weak, moderate, or strong effect, see Section 2.

**Table 2:** Characterization of pharmacological inhibitors in phenotypic assays.

Chemical	Target	PBMC	Primary B cell	Namalwa						
		MLR	IgG production	Viability WST-1	CD40	CD69	CD70	CD80	CD83	CD86
Bortezomib	Proteasome: 30–100	35	90	10	—	—	—	—	—	—
Ibrutinib	BTK: 0.5	4 000	<1	1 800	690	↗	670	>3 000	730	<30
Chloroquine	TLR 3, 7, 8, and 9: 560	>10 000	95	>10 000	70	90	1 000	60	210	<30
Dasatinib	SRC: 0.55 BCR/ABL: 3 LYN: 8.5 Other TK	15	55	20	—	—	—	—	—	—
LY-294,002 hydrochloride	PI3K: 3 000	5 100	670	4 600	2 800	>3 000	2 500	2 200	>3 000	450
MK-2206 2HCl	AKT1/2/3: 8/12/65	3 500	25	2 700	1 500	↗	1 200	1 100	120	<30
AZD-5363	AKT1/2/3: 3/7/7	2 600	600	3 000	1 500	↗	850	>3 000	2 200	<30
TPCA-1	IKK-2: 17.9 IKK-1: 400	360	450	2 800	210	550	480	700	550	<30
STAT3 inhibitor VII	STAT3: 170	8 500	>10 000	>10 000	>3 000	>3 000	>3 000	2 800	>3 000	1 000
SAHA	Class I & II histone deacetylase (HDAC): <86 HDAC1: 13.7	5 000	440	2 100	>3 000	↗	>3 000	1 300	>3 000	>3 000
Mirin	Mre11-Rad50-Nbs1 (MRN) complex (DNA-repair) Cell-free: 12 000 Human: 66 000	>10 000	1 400	>3 000	>3 000	>3 000	>3 000	>3 000	>3 000	>3 000

The table shows the IC_50_ (nM) of the inhibitors on the molecular target and in the different phenotypic assays: MLR with human PBMCs, IgG production by human B cell stimulated by ODN2006, and surface markers expression on ODN2006-stimulated Namalwa. Cytotoxic counterscreen (WST-1) was performed on Namalwa cells. ↗: indicates an increase in expression.

## Abbreviations

BCR: B cell receptor
BTK: Bruton's tyrosine kinase
CD: Cluster of differentiation
DMEM: Dulbecco's Modified Eagle's Medium
FCS: Foetal calf serum
Ig: Immunoglobulin
IKK: I*κ*B kinase
IL: Interleukin
LPS: Lipopolysaccharide
MFI: Mean fluorescence intensity
MHC: Major histocompatibility complex
MLR: Mixed lymphocyte reaction
NF-*κ*B: Nuclear factor kappa-light-chain-enhancer of activated B cells
ODN: Oligodeoxynucleotide
PBMC: Peripheral blood mononuclear cell
PBS: Phosphate buffered saline
PI3K: Phosphatidylinositol-4,5-bisphosphate 3-kinase
STAT3: Signal transducer and activator of transcription 3
TLR: Toll-like receptor
TNP: 2,4,6-Trinitrophenyl hapten.
